# Exploring the relative contributions of reward-history and functionality information to children’s acquisition of the Aesop’s fable task

**DOI:** 10.1371/journal.pone.0193264

**Published:** 2018-02-23

**Authors:** Elsa Loissel, Lucy G. Cheke, Nicola S. Clayton

**Affiliations:** Department of Psychology, University of Cambridge, Cambridge, United Kingdom; Kyoto University, JAPAN

## Abstract

Investigation of tool-using behaviours has long been a means by which to explore causal reasoning in children and nonhuman animals. Much of the recent research has focused on the “Aesop’s Fable” paradigm, in which objects must be dropped into water to bring a floating reward within reach. An underlying problem with these, as with many causal reasoning studies, is that functionality information and reward history are confounded: a tool that is functionally useful is also rewarded, while a tool that is not functionally useful is not rewarded. It is therefore not possible to distinguish between behaviours motivated by functional understanding of the properties of the objects involved, and those influenced by reward-history. Here, we devised an adapted version of the Aesop’s Fable paradigm which decouples functionality information and reward history by making use of situations in which the use of a particular tool *should have* enabled a subject to obtain (or not obtain) a reward, but the outcome was affected by the context. Children aged 4–11 were given experience of a range of tools that varied independently in whether they were functional or non-functional and rewarded or non-rewarded. They were then given the opportunity to choose which tools they would like to use in a test trial, thereby providing an assessment of whether they relied on information about functionality or the reward history associated with the object or a combination of the two. Children never significantly used reward history to drive their choices of tools, while the influence of functionality information increased with age, becoming dominant by age 7. However, not all children behaved in a consistent manner, and even by 10 years of age, only around a third exclusively used functionality as a basis for their decision-making. These findings suggest that from around the age of 7-years, children begin to emphasize functionality information when learning in novel situations, even if competing reward information is available, but that even in the oldest age-group, most children did not exclusively use functionality information.

## Introduction

“Causality is a constraint common to all ecological niches” ([1], p.645). All environments, however diverse, contain cause-effect relationships. Being able to detect and use these causal links to predict and manipulate surroundings is therefore highly adaptive. Much research on how individuals understand these relationships concentrates on causality in the domain of folk physics, often using means-end or tool-use methodologies.

Two and a half thousand years after Aesop’s classic fable of the thirsty crow dropping stones into a pitcher to raise the level of the water inside, the “Aesop’s fable” paradigm has become a popular means by which to assess causal cognition in birds and children (e.g. [2, 3–6]). In this paradigm, subjects need to drop sinking objects into a tube filled with water, causing the water level to rise and bringing a floating reward within reach. While most subjects tested proved capable of item-dropping behaviours, variants of the task were developed to explore what individuals know and learn about water displacement. These tests have mainly fallen into two categories: substrate-choice tasks, which explore the importance of functional context, and object-choice tasks, which explore the importance of functional tools.

In substrate-choice tasks, individuals are presented with two tubes. One is filled with water, with a reward floating on the surface. The other tube has an identical reward at the same level, but is either filled with a non-displaceable substrate (e.g. sawdust or sand [2]) or is empty, with the reward attached to the inside of the tube (“air-filled” tube [7]). To obtain a reward, individuals must therefore choose to drop sinking objects into the functional (i.e. water-filled) tube over the non-functional one [8]. In object-choice tasks, individuals are presented with a single tube of water and a selection of items to choose from. Half the objects are “functional”: they sink and displace enough water to cause the floating reward to rise. The other half are less- or non-functional: they will either displace significantly less water, or none at all. Individuals should therefore choose to drop the functional objects in order to retrieve the reward (e.g. [2, 7, 9]).

Successful performance on causal tests such as the Aesop’s fable task suggest some comprehension of cause and effect. However, this can take different forms. An organism can either recognise a link between cause and effect (i.e. they can know *tha*t x causes y), or they can understand that a *mechanism* ties cause and effect (i.e. they know *why* x causes y). The distinction between these two levels of cause-effect understanding can be considered as the difference between associative learning and causal reasoning.

Associative learning is the process by which a stimulus becomes associated with an outcome when the two co-occur with both high temporal contiguity and high contingency (e.g. [10]). This learning system has evolved as a simple and reliable mechanism to allow an individual to react to reliable co-occurring events in the environment. It is therefore a highly efficient and effective cognitive process with which to learn about cause-effect relationships (e.g. [11]). In contrast, individuals employing causal reasoning represent causal relationships not just by the co-occurrence of two events (or action and outcome), but as a *causal* event or action and a *resulting* effect. This form of reasoning involves the understanding that a *mechanism* exists by which the cause leads to the effect, and at a more sophisticated level, what that mechanism is.

On a cognitive level, this form of causal understanding is very different from that seen in associative learning: predictions of outcomes are based on a model of how the world works, rather than statistical regularity [12]. In theory, the two can also produce distinct behaviours. Individuals that know *that* a cause leads to an effect will be able to act appropriately should that exact situation re-occur in the future. However, subjects that understand *why* a cause leads to an effect will be able to generalise their learning to other situations based on the same mechanism. Still, in practice, it is often very difficult to assess exactly what has been learned. In the vast majority of causal scenarios, the effect closely follows the cause (contiguousness). Cause and effect are also highly contingent: one is unlikely to occur in the absence of the other. This makes it difficult to disentangle whether the subject has formed an associative link between cause and effect, and are simply likely to repeat an action that has previously been rewarded, or whether they understand *how* these two factors are linked and thus rely on information about the current functionality of an object.

This limitation is especially pertinent in tool-use studies—including the Aesop’s fable paradigm. When a tool is used by an individual to successfully obtain a reward, the subject can acquire two types of information. First, that this tool is rewarded, i.e. *that* it leads to reward. Second, that this tool is “functional”, i.e. *why* it leads to reward. In the vast majority of laboratory studies on causal reasoning, the functional items are also reliably those that are linked to reward. Since information about functionality is confounded with reward history, it is impossible to differentiate between function and reward-history-based learning.

In the Aesop’s Fable task, associative learning and causal reasoning are confounded: the objects that are functional are also the ones whose use is consistently linked with reward. These objects may therefore become attractive for use by virtue of mere association with reward, rather than being chosen for their utility. One way in which researchers have attempted to distinguish between causal reasoning and associative learning is by analysing only the first trial of an individual’s first exposure to a problem [13]. This is a useful method to assess pre-existing knowledge or understanding, but brings problems of its own. First, if an individual chooses an appropriate tool on their first trial, the contribution of previous learning to this performance is unknown. It remains possible that associative learning from previous experiences in highly similar situations has been generalised to this new context [14]. Second, if an individual fails to choose an appropriate tool on their first choice, this is not necessarily informative as to their capacity to understand the relevant functionality. At most, one can conclude that, in the context of this particular choice, the subject does not *already* have such an understanding. By not allowing repeated exposure to the problem, such experiments only partially rule out the influence of previous reward history, while completely precluding the influence of learning about the physical rules involved.

In order to investigate understanding of functionality we must therefore differentiate causal reasoning from associative learning based on reward history, while also recognising the importance of learning and experience in the formation of causal understanding. This requires exposing individuals to situations in which functional items *do not* result in obtaining a reward, while non-functional items do so. These situations arise when external circumstances interfere with the link between the functionality of a tool and the receipt of a reward. Examples of these types of experiences are in fact relatively common in everyday life. One can have a tool that is usually functional (for example, a metal hammer) that does not achieve the desired outcome because of the context of use (a bent nail) but *would have* been successful in more standard circumstances. Meanwhile one can have a usually non-functional tool (an inflatable hammer) that succeeds in achieving the desired outcome because of the context of use (for example, a nail going into a large, pre-drilled hole) but *would not* have been successful in more standard circumstances. In these circumstances, a “functionality learner” would learn about the functional properties of these tools through experience (the metal hammer exerts a large percussive pressure on the nail, the inflatable hammer exerts very little pressure). On the other hand, a “reward-history learner” would learn the reward associations. If both individuals were later given a choice of the two tools in a “standard” context, this same learning environment would predict different choices.

The current experiment was designed to assess the relative contribution of causal reasoning and associative learning to choices in an Aesop’s fable task in 4–11 year old children. In particular, we examined how the influence of these two competing approaches may shift as the children get older. To investigate this, the experience of being rewarded for using a particular tool was uncoupled from whether or not this tool was functional. For example, using a functional tool (i.e. an object that sinks) could lead to the experience of not being rewarded (the context prevented the water level from rising). Conversely, using a non-functional tool (i.e. an object that floats) could lead to obtaining a reward (serendipitously provided by the context). These experiences give information as to the functional properties of the tools (the children can observe that one object sinks and displaces water), but in a manner that does not confound this information with reward history. Which objects children then choose to use in a transfer task, where the context was more standard, would indicate whether their experience with the tool taught them information about the tool’s functionality, or the tool-reward association. From the pattern of performance obtained across a number of situations varying in reward and functionality information, it should be possible to statistically model the relative contribution of reward- and function-based information to children’s choices. In a separate analysis, we can also identify what proportion of children of different ages are exclusively using a specific type or combination of information to guide their choices.

Previous research suggests that children are capable of forming an understanding of cause and effect from a very young age. Infants from 8 months can form expectations from complex statistical regularities [15–17], and are able to act on such information in a goal directed manner from 2.5 years of age [18]. Children of 3–4 years are furthermore able to explicitly identify action-outcome relationships from covariation and contiguity when no obvious mechanism of action is shown [19–22]. It is therefore likely that children of at least 4 should be able to learn to identify a successful course of action using associative learning. However, while performance improves gradually from around 5-years, children do not reliably pass a number of complex tool using tasks, including the Aesop’s fable, until the age of around 8–10 years [3, 23–27]. This may imply that these tasks involve more complex levels of associative learning than those previously used. Such an account would suggest that the gradual increase in performance with age in the Aesop’s fable task seen in previous studies [3, 23] may reflect a growing sophistication in associative learning. In the current study, this would predict that right from the youngest age children would rely on association with reward to guide their choices, becoming more successful with age. This pattern would be observed as a consistent tendency for statistical models emphasizing reward-information to be the best fit with children’s behaviour, with the degree of fit increasing with age.

Alternatively, it is possible that the type of information used to solve the Aesop’s fable task may change over time. Cheke and colleagues [3] asked children to describe what they thought was happening in a modified version of the test. The youngest participants mostly offered no explanation at all. However, the proportion of individuals able to describe the cause-effect relationship (i.e. state *that* cause led to effect) remained relatively stable from around the age of 5. At around 7-8-years, descriptive explanations became outweighed by attempts at mechanistic explanations (i.e. statements of *why* cause led to effect). While the precise mechanism given by the children was not always correct, the effort to provide such an account suggests that participants of this age may be emphasizing functionality-based information in their learning. It is therefore possible to hypothesize that while the youngest children (e.g. 4–5 years) may be unable to identify cause-effect relationships in the Aesop’s fable task, children of intermediate age (5-7-years) may rely on a reward-based associative information. This in turn would be replaced by choices driven by functionality information around the age of 7 and upwards. In the current study, this would be observed as a change with age in the ability of different statistical models to fit children’s behaviour. In the youngest children, no model would significantly fit the data, then models emphasizing reward and functionality information would best predict behaviour in intermediate and older children respectively.

## Materials and methods

### Ethics statement

This study was approved by the University of Cambridge Psychology Research Ethics Committee (pre.2013.109) and conducted under the European Research Council Executive Agency Ethics Team (application: 339993-CAUSCOG-ERR). Informed written consent was given by the parents of each child before they took part in the experiment.

### Participants

189 children aged 4 to 11-years were recruited from five schools around Cambridgeshire during the 2014–2015 school year. Six subjects (3 males age 4, 5 and 11; 3 females age 9, 5 and 6) were excluded from the sample due to experimenter error (N = 4), language barrier (N = 1) or a diagnosis of autism (N = 1). The remaining 184 children (97 boys) were included in the analysis (4-year-olds: n = 21; 5-year-olds: n = 31; 6-year-olds: n = 35, 7-year-olds: n = 34, 8year-olds: n = 19, 9-year-olds: n = 19, 10-year-olds: n = 19, 11-year-olds: n = 5). Given the small sample of 11-year-olds, these data were combined with the 10-year-olds for analysis.

### General procedure

Participants were tested at local schools in a separate room or quiet area, and presented with a series of tasks involving water displacement. They received one session that lasted approximately 30 minutes.

The experiment was structured into three phases. The training phase familiarised children with the materials and concepts of the experiment. The exploration phase allowed them to manipulate a range of objects in the context of a water task. These objects were either functional or non-functional in terms of displacing water, and their use was either rewarded or unrewarded (depending on the container used). During this phase, children were therefore able to learn information about functionality and reward about each object in isolation. Finally, the choice phase allowed the children to choose between specific object pairings in order to pick which objects to use in a neutral context.

### Training phase

During the training phase, participants were introduced to “tokens”. These were made of cork and metal such that they floated in water but were also magnetic. The children were told that if they could remove the tokens from inside of a toy they could be exchanged for stickers. The token was first inserted into a “platform apparatus”–a Perspex box containing a platform held in place by a magnet. Upon dropping a heavy object into the apparatus through a vertical Perspex tube, the platform–as well as the token it supported–was released [2]. Participants were shown how to obtain the token and encouraged to try themselves. A slightly modified version of the platform apparatus was then given, where the platform was physically (and obviously) prevented from being triggered by means of a cardboard wedge. Children were still prompted to insert objects inside the box to try to get the token out. Upon failure to obtain the reward they were told that “sometimes things don’t work”.

The magnetic properties of the token were then demonstrated to the children by the introduction of a “fishing rod”. The fishing rod was an 9x6cm plastic square attached to a 6cm string glued to a magnet. Subjects were shown that the token can “stick” to the fishing rod and subsequently encouraged to use it to extract the token floating in a container full of water.

### Exploration phase

#### Materials: Containers

**Fig 1:**
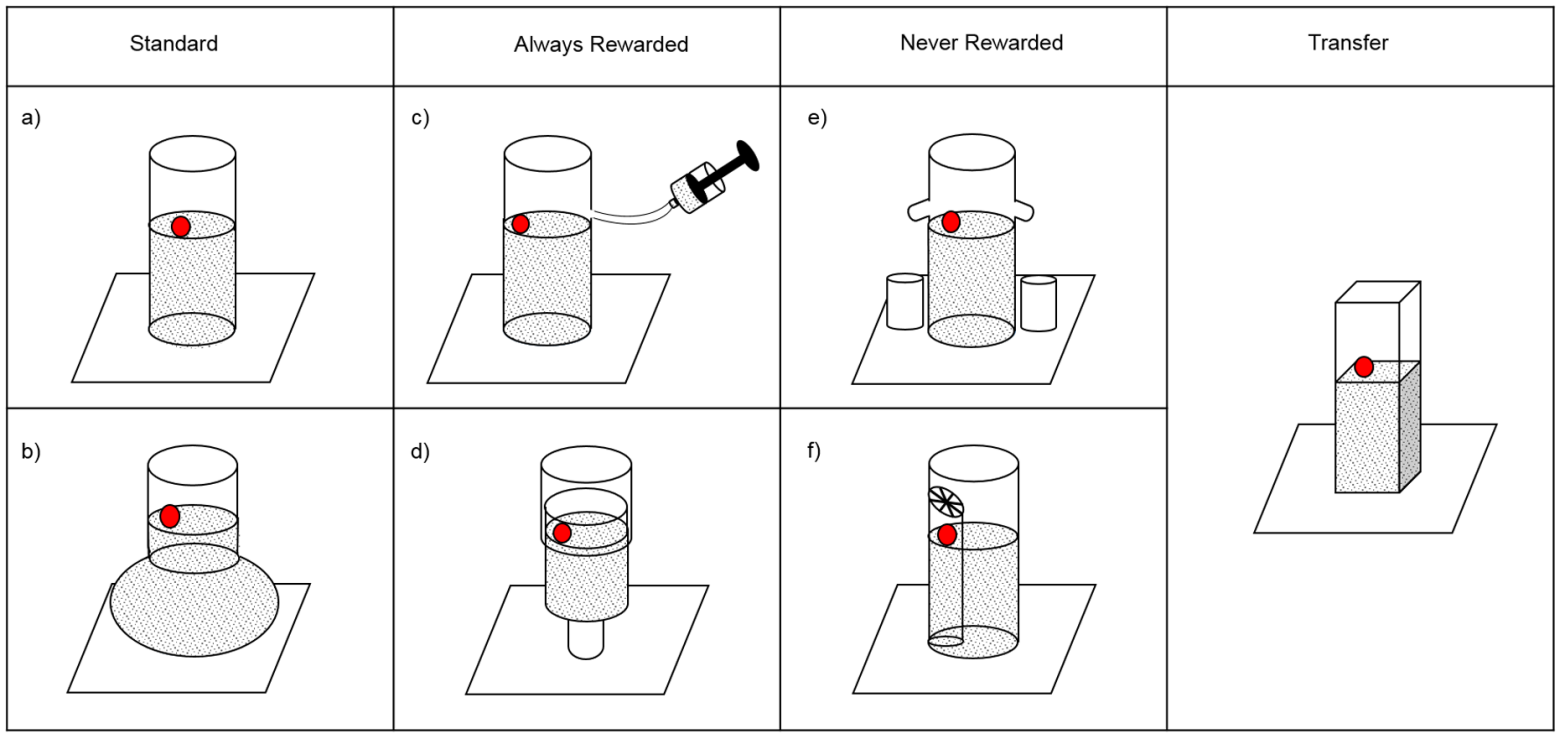
Containers used during the exploration and choice phases. Far left: Standard containers in which functional objects lead to reward, and non-functional objects do not: (A) the “Tube” and (B) the “Flask”. Centre left: Always-Rewarded containers which will lead to reward regardless of the functionality of the objects: (C) The “Syringe” in which water is added through a syringe; (D) The “Piston” in which the external sleeve is pulled down, bringing the aperture closer to the water level. Centre right: Never-Rewarded containers which will never lead to reward regardless of the functionality of the objects; (E) The “Leaking Tube” in which the water overflows upon each object insertion; (F) The “Sectioned Tube” in which the token is trapped in a smaller internal tube. Far right: The “Transfer” tube, a rectangular container.

Each element of the exploration phase involved a Perspex container filled with water to a level 8cm below the aperture, with a token floating on the surface of the water. All water containers were attached to a 10x10cm Perspex base, were 18cm tall and had an inner diameter of 5cm at the open aperture. In each container, the water was coloured to match the colour of the object it was used with, to facilitate the children’s memory for the pairing. There were 3 types of container: ‘standard’, ‘always-rewarded’ and ‘never rewarded’. For counterbalancing reasons, there were two versions of each type of container (Fig 1).

Standard containers were containers in which functional objects would lead to reward, and non-functional objects would not. The standard containers were either a “Tube”—a clear Perspex cylinder—or a “Flask”, a clear laboratory-style flask with a spherical base (maximum diameter: 11cm) and cylindrical ‘neck’ (length: 12cm). For the “Flask”, the level of the water was always above the level of the base, such that although this container contains more water in total, insertion of a functional object would cause the level to rise by the same amount as in a plain tube.

Always-Rewarded containers were containers which would lead to reward regardless of the actual functionality of the objects. The “Syringe” was a Perspex tube connected on its side to a 50cm flexible silicon piping linked to a 100ml syringe. When the syringe was manipulated by the experimenter, extra water was inserted into the tube. The “Piston” was formed of two Perspex tubes (internal diameters of 5cm and 4.5cm respectively) tightly fitting into each other. The inside tube contains water while the outside one acts as a tightly fitted sleeve that can be moved up and down. Despite their equal length, in the initial position the tubes are not lined up: instead, the sleeve sticks out by 10cm, bringing the height of the apparatus to 18cm and the distance from the top of the apparatus to the water-level to 8cm. Manipulation of the outer tube by the experimenter would incrementally reduce the distance between the water-level and the aperture by bringing the tube-aperture closer to the level of the water.

Never-Rewarded containers were containers which would never lead to reward regardless of the functionality of the objects. The “Leaking Tube” was a Perspex cylinder with two holes on the side placed just above the initial water level. Two small silicon pipes were inserted in these holes, allowing any water above that level to flow out freely. Two small cylindrical containers stand under the piping, collecting the water as it overflows. Raising the water level in this container would not bring the water level closer to the tube aperture since all displaced water would leak away, causing the water level to return to its original position. The “Sectioned Tube” was a standard 5cm diameter Perspex cylinder containing a smaller 2x12cm tube topped by a lid. The token was placed within the smaller tube, meaning that raising the water level of the main tube would not move the token.

#### Materials: Objects

The objects were either functional (displaced water) or non-functional (did *not* displace water). All objects measured approximately 2x2cm and were of comparable weights; however, they were of different shapes and colours to facilitate discrimination. For counterbalancing reasons, four versions of each type were created.

Two types of non-functional objects (F-) were presented. Hollow objects were created using a frame of wire covered in a thin layer of polymer clay. These items would sink but only displace a negligible amount of water since the water could flow through. Floating objects were made from polystyrene. There were four non-functional items in total, varying in type, shape and colour (blue hollow cube, green hollow cube, red floating cylinder, yellow floating cylinder). The variety in type (hollow / floating) and appearance (colour / shape) of the objects was created in order to control for any pre-existing preferences that would affect performance. By counterbalancing appearance and type of object, we were able to infer that any consistent pattern of behaviour would be due to the functionality of the object, rather than other factors.

All functional objects (F+) were made of polymer clay displacing the water by 0.4cm. The four unique functional object types varied in shape and colour (red solid cube, yellow solid cube, green solid cylinder, and blue solid cylinder). This variety was created to match the variety present in the non-functional objects.

#### Procedure

**Fig 2:**
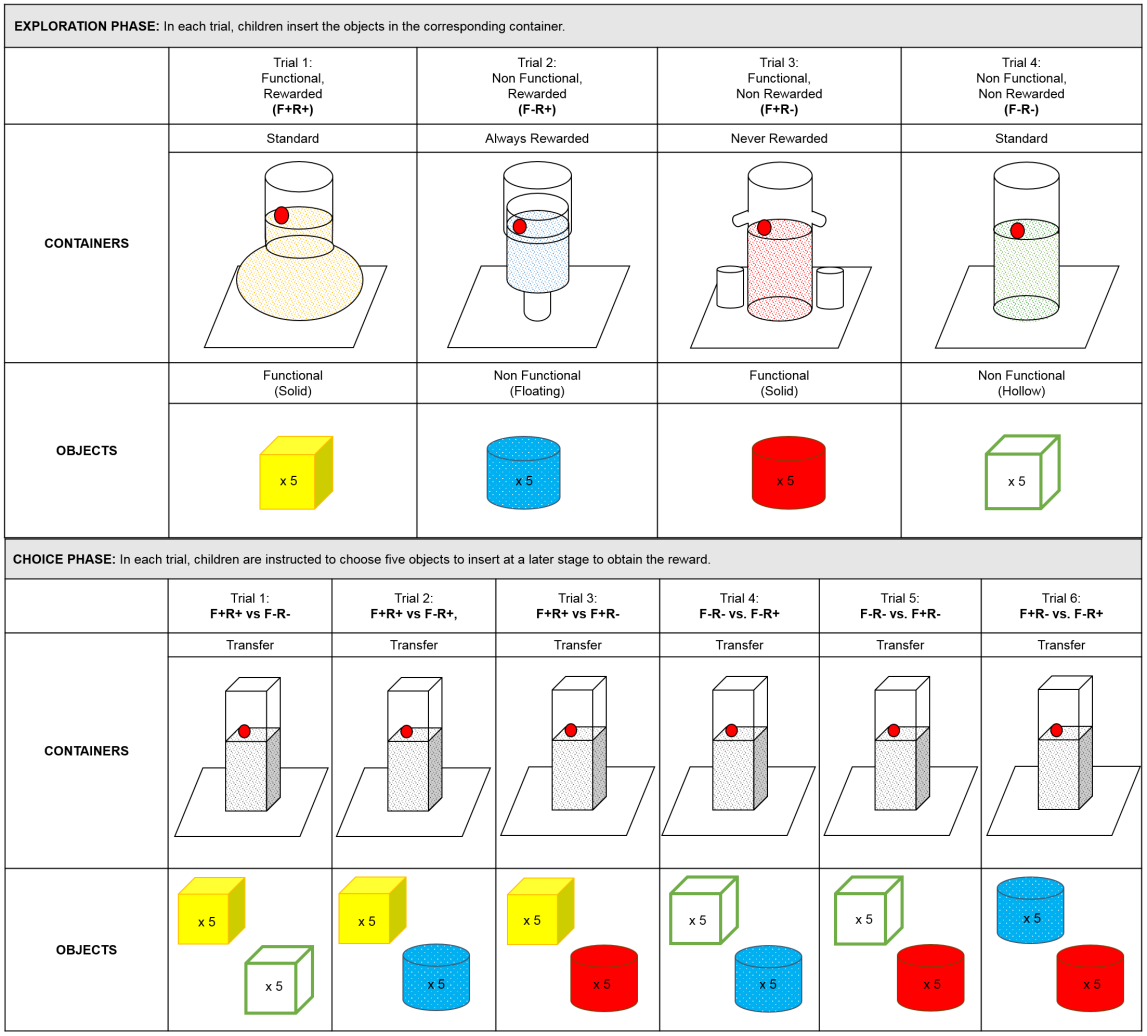
Exploration and choice phases. (A) The exploration phase: Children experienced the four different types of objects (F+R+, F-R+, F+R-, F-R-) through four consecutive exploration trials. In each trial, five objects are inserted in the corresponding container, either leading to the acquisition of the reward or not. Each object-container pair has the same colour. (B) The choice phase: Children have six choices as to which previously experienced objects to use with the transfer tube: F+R+ vs. F-R-, F+R+ vs F-R+, F+R+ vs F+R-, F-R- vs. F-R+, F-R- vs F+R-, F+R- vs F-R+.

The exploration phase gave the opportunity for children to experience four different function/reward conditions once each (F+R+, F-R+, F+R-, F-R-) through four consecutive exploration trials (Fig 2). Children were split into four groups for counterbalancing purposes. These groups experienced different versions of the objects and containers, and in different orders, but otherwise went through identical conditions.

The Functional/Rewarded (F+R+) condition paired the functional objects with standard containers, leading to reward. The Functional/Non-rewarded (F+R-) condition associated functional objects with never-rewarded containers. The Non-functional / rewarded (F-R+) condition coupled non-functional objects with always-rewarded containers. Finally, the Non-functional/Non-rewarded (F-R-) condition combined non-functional objects with standard containers, and did not lead to reward. Each exploration trial took place as follows: the children were given the fishing rod, one type of water container, and one set of objects. Five objects were provided, all of the same type, colour and shape: the colour of the water matched the colour of the object. The token was dropped into the water by the experimenter and children were given one minute to retrieve it. The starting level of the water was always too low for the token to be retrieved immediately with the fishing rod. If they did not do so spontaneously, the children were encouraged to insert the provided objects in the container. Children were advised to insert all five objects regardless of the outcome of the first insertion. In rewarded trials, insertion of 5 objects was required to bring the token within reach of the fishing rod. In non-rewarded trials, no quantity of objects would ever bring the token within reach of the fishing rod. After each trial, children were shown a card displaying the container and object they had just used, with a representation of the token either added or not, to represent whether this combination had led to reward. This card was then visible throughout the remaining exploration and choice trials.

### Choice phase

#### Materials

The transfer tube was an 18cm tall square container (4x5cm) filled with clear water up to a height of 8cm from the aperture. The objects given for the children to choose between were identical to the ones they used during the exploration phase (Fig 1).

#### Procedure

Once all four trials of the exploration phase were completed, the children entered the “choice” phase. This started with the presentation of the “transfer tube”, a novel container filled with clear water. A token was placed in this container, and it was demonstrated to the children that it was out of reach.

The choice phase contained 6 unique trials opposing two types of objects. Each choice–referred to hereafter as choice-trials—contrasted different combinations of functionality and reward history: (1) F+R+ vs. F-R-, (2) F+R+ vs F-R+, (3) F+R+ vs F+R-, (4) F-R- vs. F-R+, (5) F-R- vs F+R-, (6) F+R- vs F-R+.

Each trial followed the same structure. The transfer tube with the token was presented, along with the fishing rod. Alongside were given two types of objects (five of each). The children were instructed to choose five of the 10 objects to use ‘later’ to try to obtain the token from the transfer tube. The selected objects were put on the side in an egg box. Importantly, the use of the selected objects did not take place immediately after selection, so as to prevent any learning/feedback that would influence the following choices. Each choice-trial was unique and no choice was repeated.

Once they had made all six choices, children had the opportunity to try to retrieve the token six times using the objects that they have previously selected.

At the end of the choice phase, the children collected the stickers that they had won during the experiment and were instructed not to discuss the “game” they had been playing with their classmates.

### Analysis

#### Structure of the data

For each child and for each of the six choice trials, the number of each type of objects chosen by the children was recorded. Each of these would be out of a possible total of 5.

#### Models

We defined five different models representing distinct patterns of behaviour. For each model, we highlight a particular type of information, or combination of information-types, that could drive behaviour, and predict which objects should be chosen if relying solely on this information and choosing perfectly. As such, these five models give predictions as to what a theoretical child would choose if they were basing their decisions purely on reward-history, functionality information, or a particular combination of the two.

By comparing the actual choices made by the participants to the predictions for each model, we can calculate a degree of fit for each model to observed behaviour. By comparing the relative fit of different models, we can thereby assess which model/s provide the best statistical fit for the children’s choices. This should allow us to infer which informational factors (reward-history or functionality) most contribute to decision-making in this task. It is not possible to directly compare the model predictions to each individual choice made by each child. However, we can estimate how well that child’s pattern of choices across the 6 unique choice-trials fit each model’s predictions. To do so, we establish a reference point for each model: A ‘perfect’ score obtained by only selecting the objects based on that model. Then we assess the degree to which the score obtained by the children fits with that reference point. The degree of fit gives an indication of the consistency with which this information influences behaviour. It is possible for a particular model to be the best fit of the 5 available models, but still have a low level of fit. This would suggest that the child’s decision-making is not *consistently* reliant on any particular type of information, but that one type of information has *more influence* than the others. Conversely, if a child’s behaviour has a very high level of fit with a particular model, this may suggest that the child is explicitly relying on a particular form on information, or following a strategy with a specific weighting of both types of information (such as, rely on functionality information where it is available, where it isn’t, go with whatever was previously rewarded).

Different models emphasise different factors or a combination of factors that may influence children’s choices. The ‘Function Only’ model (F) considers *only* functionality information. A child 100% fitting this model would take into account *only* whether or not the objects are functional in the context of a water displacement task. Whether using the object has previously led to actually obtaining a reward would be disregarded. In choices where both types of items share the same functionality, the participant would choose at random. The ‘Reward-Only’ model (R), considers *only* reward information. A child 100% fitting this model would only take into account the experience of having been previously rewarded for using an object. Whether the objects are functional in the context of a water displacement task would be disregarded. In choices where both types of items share the same reward history, the participant would choose at random. The ‘Function then Reward’ model (F -> R) gives precedence to the object’s functionality over the experience of having been rewarded. A child 100% fitting this model would always choose the more functional object where this choice was available. However, when given a choice between two equally functional or non-functional objects that differ in their reward history, the previously rewarded object would be preferred. The ‘Reward then Function’ model (R -> F) prioritises the experience of having been rewarded over object functionality. A child 100% fitting this model would always choose the previously rewarded object where this choice was available. However, when given a choice between two equally rewarded objects which differ in functionality, the functional object would be preferred. Finally, the ‘Causal Link’ model (CL) predicts that the child does not rely on functionality or reward history alone but instead on how familiar they are with the relationship between insertion and reward outcome. In two of the four conditions (F+R+ and F-R-) that use standard containers, the relationship is not affected by outside influence and therefore might be considered more ‘expected’. Solid, sinking objects raise the water, making it easy to reach the token; hollow items or floating props do not displace liquids, leading to an unrewarded trial. However, in the other two conditions (F-R+ and F+R-) using always-rewarded or never-rewarded containers, the link between item insertion and reward outcome is influenced by external factors and therefore possibly less familiar and straightforward. Instead, the outcome is tied to the intervention of the experimenter or the design of the container. It is possible that in the F+R+ and F-R- conditions, participants may be able to form an understanding of the objects and to predict how they will behave in the transfer tube used in the choice phase, using previous experiences to guide their judgements. However, it is conceivable that such understanding and prediction couldn’t be formed for the items used in F+R- and F-R+ conditions, leading the children to be uncertain of the consequences of their insertion. A child 100% fitting this model therefore would therefore rely on actively choosing F+R+ and rejecting F-R- whenever possible, but in cases where F+R- objects are opposed to F-R+ items, the participant would choose at random.

#### Data preparation

The reference score for each model (the perfect score obtained only by selecting the objects based on that model) was created through three steps. First, we establish which choice trials should be considered for each model (Table 1). If the model predicts random choice for a given choice trial (i.e. a trial that opposes two objects that are equally likely to be picked according to this model, such as two previously rewarded objects for the “reward-only” model, see Table 1), this trial isn’t taken into account in the establishment of the reference score. This is because the model cannot predict what “random choice” might be for each individual. Then, for each choice trial, we determine which type of objects *should* be chosen according to the model. For each choice, this type of object is given a score of 5/5. Finally, the scores are summed across all the appropriate choice trials to give the reference score. Each choice-trial therefore contributes specific information to the reference score, without duplication or redundancy. Each model possesses its own reference score, which is to be compared to the actual choices made by the children.

**Table 1:** Predicted choices for each model.

CHOICE	FUNCTION ONLY (F)	REWARD ONLY (R)	FUNCTION THEN REWARD (F -> R)	REWARD THEN FUNCTION (R -> F)	CAUSAL LINK (CL)
**F+R+ VS F-R-**	F+R+	F+R+	F+R+	F+R+	F+R+
**F+R+ VS F+R-**	*Random- Not considered*	F+R+	F+R+	F+R+	F+R+
**F+R+ VS F-R+**	F+R+	*Random- Not considered*	F+R+	F+R+	F+R+
**F+R- VS F-R+**	F+R-	F-R+	F+R-	F-R+	*Random- Not considered*
**F+R- VS F-R-**	F+R-	*Random- Not considered*	F+R-	F+R-	F+R-
**F-R+ VS F-R-**	*Random- Not considered*	F-R+	F-R+	F-R+	F-R+

To do this comparison, for each child and for each of the five models, an “observed” score is created based on the actual objects chosen by the participant, with regard to the predictions of the model. The observed score for each child and each model is created using the same choice trials used to establish the reference score for that particular model. As such, we do not consider behaviour in trials where the model would predict random choice. For each trial, we identify how many of the items predicted to be chosen by the model were in fact chosen by the child. For example, if the model predicts for a particular choice that the functional item should be chosen, and a child chooses 3 of the functional items and 2 of the non-functional items, the observed score for that choice would be 3/5 for that model. These scores are then summed across all relevant choices for that model. This process produces 5 observed scores for each child–one for each model.

For each model, observed scores are then compared to the reference scores to give a measure of model-fit. This measure quantifies how well the participant’s behaviour is consistent with that predicted by the model. A score of 100% means that a child chose all the objects expected to be picked according to that model. In the example given above, the child would have a model-fit of 60% for that model. Statistical analysis is then conducted on upon these measures of model-fit.

The models assessed are not completely independent from one another: the way the reference scores are created means that there is an overlap, especially between closely related models. For example, the choices that children are predicted to make by the “function only” model are close to the ones predicted by the “function then reward” model. This means that a child’s behaviour may be equally well described by multiple models.

Finally, the number of children whose behaviour perfectly or near-perfectly (over 95%) fit each model was recorded. These children might be said to be relying exclusively on a particular form of information. While the general model-fit analysis will assess the relative influence of different types of information on choices statistically, this analysis will explore how many individual children are committed to a single information type or combination. This will allow for differentiation between situations in which, for example, 100% of children are 50% influenced by reward history, or whether 50% of children are 100% influenced, while 50% of children are not influenced at all.

## Results

Children’s scores were normally distributed, as assessed by Shapiro-Wilk’s test, except for the “Function only” (p = 0.02) and “Reward only” (p = 0.028) models in 9 year-olds and the “Function only” model in 10 year olds (p = 0.03). Where data were normally distributed, parametric tests were conducted.

### Are children’s choices distinguishable from random?

**Fig 3:**
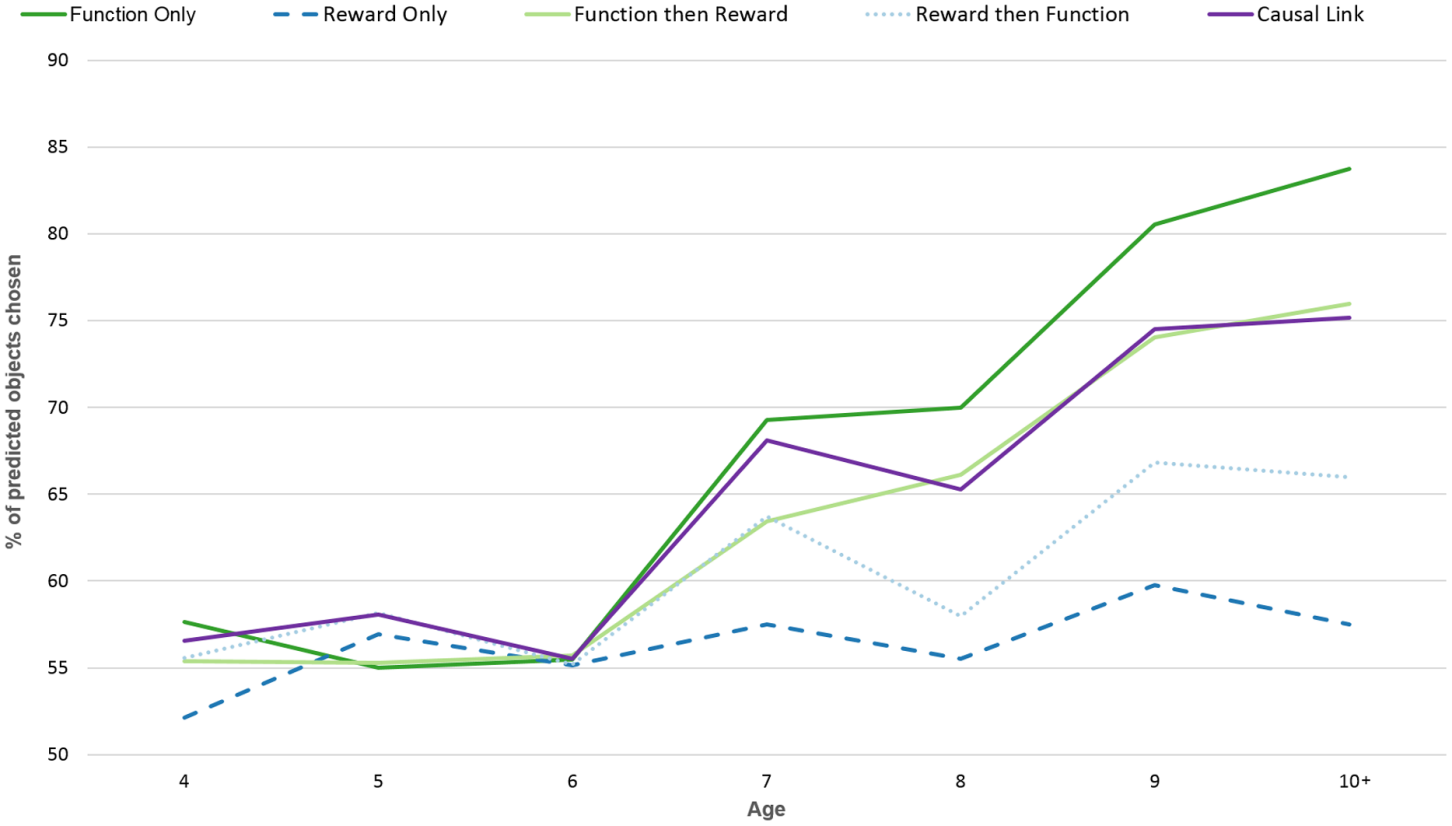
Percentage of objects chosen that fit with each model.

The first analysis explores whether or not children in each age group made choices in a way that is distinguishable from random. This will also validate our model-based approach to data analysis. One sample t-tests were used to compare the model-fit of each age group with each model to chance (50%). The results indicate that behaviour of the four-year olds cannot be distinguished from random (Fig 3). However, older children all demonstrated behaviour that fit with at least one model (Table 2).

**Table 2:** Difference from random choice for each model across age groups.

Model	4-YO	5-YO	6-YO	7-YO	8-YO	9-YO	10+-YO
**F**	t(20) = 1.759, p = 0.094	t(28) = 1.593, p = 0.1222	t(32) = 2.354, p = 0.024	t(33) = 6.193, p<0.001	t(18) = 4.256, p<0.001	Wilcoxen p<0.001	Wilcoxen p<0.001
**R**	t(20) = 0.585, p = 0.565	t(30) = 2.238, p = 0.033	t(31) = 3.285, p = 0.002	t(31) = 3.427, p = 0.001	*t(18) = 1*.*899*, *p = 0*.*073*	Wilcoxen p = 0.022	t(23) = 3.217, p = 0.003
**F -> R**	t(20) = 1.394, p = 0.179	t(30) = 1.830, p = 0.77	t(33) = 2.447, p = 0.019	t(33) = 6.849, p<0.001	t(18) = 4.531, p<0.001	t(18) = 5.875, p<0.001	t(23) = 9.813, p<0.001
**R -> F**	t(20) = 1.539, p = 0.140	t(29) = 3.620, p = 0.001	t(33) = 2.329, p = 0.026	t(30) = 6.325, p<0.001	t(17) = 3.221, p = 0.005	t(18) = 5.016, p<0.001	t(23) = 7.66, p<0.001
**CL**	t(20) = 1.559, p = 0.135	t(30) = 2.470, p = 0.019	t(32) = 2.258, p = 0.03	t(33) = 7.429, p<0.001	t(18) = 4.216, p<0.001	t(18) = 5.875, p<0.001	t(23) = 9.275, p<0.001

Shaded cells indicate behaviour not distinguishable from random choice.

### Which model/s best fit children’s choices?

Data were analysed using a mixed-model design ANOVA with within-subject factors of model and between subject factors of counterbalancing group, age-group and gender. Mauchly’s test indicated a violation of the assumption of Sphericity (X^2^(2) = 543.623, p = 0.000); therefore, degrees of freedom were corrected using Greenhouse-Geisser estimates of Sphericity (eta = 0.330). Level of model-fit was significantly influenced by age group (F(6, 113) = 5.493, p<0.001, eta = 0.226), counterbalancing group (F(3,113) = 3.100, p = 0.030, eta = 0.076) and model (F(1.318, 148.981) = 36.029, p<0.001). In addition, there was a significant interaction between age-group and model (F(7.910, 148.981) = 5.060, p<0.001) suggesting that *which* model best fit children’s choices was different in different age-groups (Fig 3).

Paired sampled t-tests were then conducted to assess the relative fit of each model to children’s choices in each age group. As shown by the previous analysis, none of the available models provided a good fit for the behaviour of 4-year-old children. Among 5-year-olds, no model provided a significantly better fit than any other, with the exception of the “causal link” model which predicted behaviour significantly better than the “reward only” model (t(30) = -2.486, p = 0.019). Among 6-year-olds, no model provided a significantly better fit than any other. At the age of 7, all models provided a significantly better fit than the “Reward Only” model (F: t(31) = 3.268, p = 0.002; F then R: t(31) = -3.227, p = 0.002; R then F: t(28) = -3.686, p<0.001; CL: t(31) = -4.635, p<0.001). “Causal Link” was also significantly more predictive than “Reward then Function” (t(30) = -3.637, p = 0.001). However, no single model was the ‘best’ fit. At the age of 8, function based models (“Function only” and “Function then Reward”) provided a significantly better fit than reward based models (“Reward only” and “Reward then Function”; F vs R: t(18) = 2.518, p = 0.021; F vs R then F: t(17) = 2.716, p = 0.014; F then R vs R: t(18) = -2.751, p = 0.013; F then R vs R then F: t(17) = 3.341, p = 0.003). There was no significant difference between the two function-based models or between the two reward-based models. The “Causal Link” model was a better fit than the reward-based models (R: t(18) = -2.89, p = 0.009; R then F: t(17) = -4.258, p<0.0010) but not significantly different to the function based models. At the age of 9, the “Function only” model provided a significantly better fit than all other models except for “Causal Link” (F vs R: t(18) = 3.598, p = 0.002; F vs R then F: t(18) = 3.307, p = 0.003; F vs F then R: t(18) = 3.135, p = 0.005). Both the “Function the reward” and “Reward then Function” models provided a better fit than the “Reward Only” models (F then R: t(18) = -3.43, p = 0.002; R then F: t(18) = -3.726, p = 0.001) and the “Function then Reward” model was a better fit than “Reward then Function” (t(18) = 2.605, p = 0.017). “Causal Link” was a better predictor of behaviour than the reward-based models but not significantly different to the function based models (CL vs R: t(18) = -4.585, p<0.001; CL vs R then F: t(18) = -4.485, p<0.001). Finally, at the age of 10+, the “Function Only” model provided a significantly better fit to children’s behaviour than all other models. (F vs R: t(23) = 6.496, p<0.001; F vs R then F: t(23) = 4.936, p<0.001; F vs F then R: t(23) = 6.52, p<0.001; F vs CL: t(23) = 4.154, p<0.001). All models were significantly better predictors than the reward based models (R vs F then R: t(23) = -6.848, p = 0; R vs CL: t(23) = -7.839, p<0.001; R then F vs. CL: t(23) = -9.042, p<0.001). Finally, the “Reward then Function” model was a better fit than the “Reward Only” model (t(23) = -5.87, p<0.001).

These results suggest that the older the child, the closer their behaviour can be fit to a function-based model, such that by the age of 10, the best predictor of behaviour is a model that considers only information pertaining to functionality and ignores reward-history entirely.

### How many children fit the models perfectly?

The final analysis explores how many children’s behaviour was a perfect or near-perfect fit with that predicted by the models, and how this differs across age groups. To do so, we selected only children who selected 95% or more of the objects predicted by the model. Such children might be said to exclusively base their choices on a particular type or combination of information types described by one of the models. For example, their choices may be guided solely by reward history, that is, over 95% of their choices fit those predicted by the “reward only” model. We then assessed how this number of children differs according to age.

**Fig 4:**
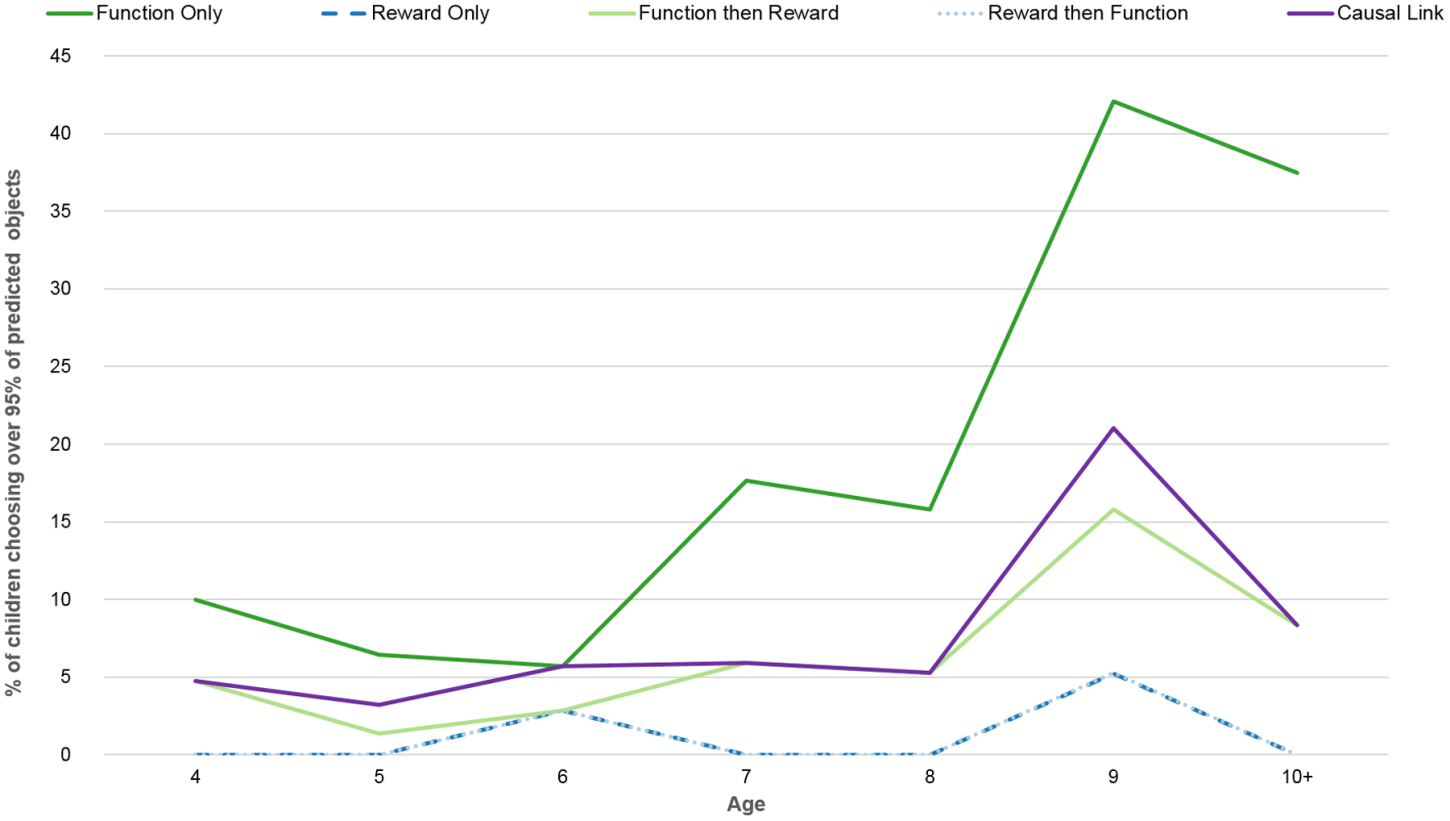
Percent of children choosing over 95% of objects predicted by a given model.

The number of children whose behaviour perfectly or near-perfectly fit at least one model significantly increased over time (Chi-Square, X^2^(6) = 223.708, p<0.001; Fig 4). The behaviour of older children was significantly more likely to perfectly fit the “Function Only” model than that of younger children (Chi-Square, X^2^(6) = 21.583, p = 0.001; Table 3). This age effect was not observed for any of the other models: Reward (Chi-Square, X^2^(6) = 5.490, p = 0.483), Function then Reward (Chi-Square, X^2^(6) = 4.564, p = 0.601), Reward then Function (Chi-Square, X^2^(6) = 5.490, p = 0.483), Causal Link (Chi-Square, X^2^(6) = 6.815, p = 0.338). Thus only functionality information significantly changed in its tendency to exclusively drive behaviour with age.

**Table 3:** Percentage of children of each age group following a given model more than 95% of the time.

MODEL	4-YO	5-YO	6-YO	7-YO	8-YO	9-YO	10+-YO
**F**	10	6.5	5.7	17.6	15.8	42.1	37.5
**R**	0	0.	2.9	0	0.	5.2	0
**F -> R**	4.8	1.4	2.9	5.9	5.3	15.8	8.3
**R -> F**	0	0	2.9	0	0	5.3	0
**CL**	4.8	3.2	5.7	5.9	5.3	21.1	8.3

## Discussion

This study aimed to explore the relative contribution of associative learning and causal reasoning to learning in the Aesop’s fable task in 4–11 year old children. Our findings suggest that the behaviour of children above the age of four can be modelled using reward and functionality information in isolation and different combinations. However, the specific model which provided the best fit for children’s choices differed between age groups. In younger participants (aged 5-6-years), there was no single model that best predicted behaviour. This may suggest that children of this age vary in how the approach the task, or that each child’s decision making is influenced by several types of information. By age 7, a pattern begins to emerge in which solely reward-based model falls behind in its ability to predict children’s behaviour. This pattern is cemented by age 8, when function-based significantly outstrip reward-based models. By age 10, the model that best fits behaviour is that which includes *only* functionality information and ignores reward information entirely.

Notably, in no age-group did the percentage of children whose choices fit a particular model by over 95% exceed 50%. This suggests that it is not the case that the majority of children in particular age-group opt for the same information-type that guides all their choices. There is, instead, a spread of information being used by children in each age-group. Nonetheless, across all age groups, the number of children opting to rely heavily on reward history (as predicted by the “reward only” or “reward then function” models) remains consistently low, while the number whose decisions rely entirely on functionality information (as predicted by the “function only” model) increases steadily with age, from around 15% of 4-year-olds to 58% in 9-year-olds 45% in 10-year-olds. This result might be taken to suggest that the change over time results from an increased tendency to rely on functionality information, rather than a decline in reward-based learning. While these figures suggest that decisions based on functionality information are the most common, this still leaves 42% and 55% of children in these respective age-groups *not* consistently relying on either type of information. While some of these are behaving in a manner consistent with the “causal-link” model, one striking change with age is the percentage of children whose behaviour does not fit any particular model. This decreases from around 80% of 4-year-olds to around 10% of 9-year-olds (although this then increases to 45% of 10-year olds, this is still lower than younger age groups). This suggests that there may be two changes that are occurring across the age range. Firstly that older children are more likely to choose exclusively according to particular information-type (although not all do so); and secondly that functionality information increases in its influence over choices as children get older, but only for around a third of children does it necessarily become the *only* type of information used.

Our findings broadly support the hypothesis that children’s behaviour starts to become markedly influenced by information about functionality when learning about cause-effect relationships at around the age of 7–8, at least in the context of the Aesop’s Fable task. This fits with previous literature suggesting that reliable performance on complex tool-use tasks emerges at around 8 years [3, 23–27]. This is also the age at which, when asked “why” something worked, children spontaneously give mechanism-based answers, rather than simply describing the cause-effect relationship [3]. That all three of these performance markers occur at the same age may suggest that first-trial success on the Aesop’s fable task requires functionality understanding. However, this does not mean that a comprehension of functionality is required to learn to perform successfully in such tasks over several trials.

The present study investigates the information emphasised by an individual after only a single trial (or 5 experiences) with each object. During the choice phase, children made 30 individual choices from 6 pairs of object-types, however they were not permitted to insert any of the objects until after all choices were made and therefore could not learn about the result of these choices. An interesting follow-up to this study would be to investigate if this information can be used to understand whether learning in a standard Aesop’s fable task requires functionality understanding in children. We know from previous research that 5–7 year olds are able to learn an Aesop’s fable task over the course of 3–5 trials [3, 23]. However, these analyses were conducted at the age-group level, and significant individual differences were evident in children’s ability to learn the task. It is possible that the type of information emphasised by an individual in the first trial of exposure may be predictive of their ability to learn a standard task over the course of several trials. To give an example, in the present study, the number of 6-year-olds whose behaviour could be predicted by a model was split relatively evenly between the different types of model. It is possible that were these same children to go on to experience 5 trials of a standard Aesop’s fable task, only those who initially emphasised functionality information would be able to learn the task. This would suggest that it is not only first-trial success that requires functionality understanding, but that learning over several trials is similarly dependant on causal reasoning. Alternatively, since any of these models could in theory produce successful behaviour (because of the confounding of function and reward in standard Aesop’s Fable paradigms), it might be that children whose behaviour fit any of the models would all learn equally, this would suggest that the same task can be learned using a variety of types of information. Finally, it is possible that added experience might lead children to alter the types of the information they use, and that this adjustment allows them to perform successfully.

One notable distinction between the older and younger children in this sample was that the behaviour of the youngest children as a group was not able to be modelled by any of the defined models, and only a low percentage of children in these younger age groups perfectly or near-perfectly fit any particular model. This may suggest that the design of the current study did not allow for sufficient experience of either functionality or reward information to allow younger children to learn. During the exploration phase, children had 5 experiences of each object interacting with water. From a functionality perspective, inserting a functional object always lead to water being displaced upwards, even if the reward ultimately did not come closer to the top. Conversely, when non-functional objects were rewarded, the mechanism by which they were so was very clearly external and not connected to the items’ intrinsic properties (e.g. addition of water through a syringe). More trials may have possibly led to an increased exposure to these cause-effect relationships, helping the younger children to form an understanding of the functionality of the objects and supporting the use of this information to guide decision making.

From a reward perspective, the amount of information regarding object-reward associations that children receive depends on the specific nature of the associative learning involved. During rewarded trials, for each object type, a single reward was received after 5 objects were inserted into the tube. A simple associative account would therefore suggest that the children had, for each object, a single experience of an object-reward association at a 5:1 ratio. By most associative accounts this would be insufficient experience to allow an association to be formed [28]. However, both Taylor and colleagues [29] and Cheke and colleagues [7] have argued that the performance of corvids in Aesop’s fable and string pulling tasks may rely on learning via perceptual-motor feedback or “incremental conditioning”. While each action (object insertion, pulling motion) does not immediately result in obtaining the reward, it does bring it closer. The authors argue that it is the approach of the reward–rather than obtaining it–that reinforces the behaviour. Indeed, when the action-reward ratio is maintained but the movement of the reward is removed, animals in such studies become unable to learn [29, 30]. In the present study, after each object-drop, the reward was either located in (or close to) its original position, or it rested detectably closer than it previously had. Even in F-R+ trials, the reward moved after ever object-drop, despite the fact that this movement was not physically caused by the object itself. The perceptual-motor feedback account would suggest therefore that in rewarded trials the children had 5 experiences of a 1:1 action-reward ratio (compared with around 25 experiences in [3]). Interestingly, recent findings by Miller, Jelbert and colleagues [31] suggest that performance of 4–9 year-old children on the Aesop’s fable task is not reliant on the movement of the reward: Learning is maintained even when the tube is opaque and movement cannot be observed. However, this does not mean that they did not rely on object-reward associations, as children were still rewarded. It may, however, suggest that any potential associative learning in this task, at least in children, is more likely to be reinforced by the receipt, rather than the movement, of the reward.

Providing extra training trials may facilitate learning in 4–7 year-old children, but it could also influence the nature of what is learned. Indeed, exploring the effect of training duration on learning could in itself be a valuable focus for future research. It is possible that increasing the number of exploration trials may lead to a preference for reward-based learning over the use of function information, especially in older children in whom both options are available. Such a dose-dependent effect of training on learning style can be seen in the progression of instrumental conditioning from goal-directed to habit-based: a small amount of training results in responding that is flexible and goal-oriented, while extended training results in responding that is inflexible and reflexive (e.g. [32]). Such an investigation would be useful not only in understanding how causal learning progresses in different circumstances, but also in identifying the best teaching strategy to promote function-based learning in school children.

That a different amount of training might skew participants towards adopting function or reward-based decisions raises the important question of whether an individual must rely on a *single* type of information in all situations. Cheke and colleagues [3] demonstrated that when presented with an “impossible” cause-effect relationship, children of around 8 years of age were able to solve the problem using reward information. Importantly, this was despite these individuals clearly *attempting* to apply functionality-based reasoning in their verbal descriptions of the task. This may suggest that children of a certain age have the flexibility to integrate or switch between the types of information they emphasise when making causal decisions. This would help explain why only a relatively small percentage of children followed a given type of information completely. However, given that older children are *able* to use reward information, the low level of fit with reward-based models, alongside the relatively strong influence of function information implies that older children may be biased their learning towards functionality information. As such, it is possible that children of younger ages are *capable* of understanding about functionality, but that what increases is a tendency to *emphasize* this information. Older children may be specifically motivated to seek functionality information when learning in new situations, while younger children may incorporate information into their learning in a less targeted manner. While “functionality” and “reward” learning have been presented here as if mutually exclusive, it is likely that any given individual could conceivably learn and be influenced by both types of information. If human adults were unable to learn reward associations in the absence of functionality understanding, we could not take advantage of reliable contingencies that are based on mechanisms beyond our understanding. As such, future studies incorporating both detectable and undetectable mechanisms would be informative as to the development of the ability not only to *use* specific types of information, but also to switch between them at need.

### Implications for non-human animals

The current research has focused on cause-effect learning in children, yet the majority of studies utilising the Aesop’s fable paradigm have been conducted with non-human animals, and in particular with members of the corvid (crow) family. As recently reviewed by Jelbert and colleagues [8], variants of the Aesop’s fable task have now been used to assess causal understanding in 4 species of corvids: Rooks [2], Eurasian jays [7], New Caledonian crows [4, 5, 9, 23] and California scrub jays [33], as well as one non-corvid bird species (great-tailed grackles [34]).

While success rates vary between species and across specific tests, birds’ performance on both substrate-choice and object-choice Aesop’s fable tasks suggest an impressive ability to learn which option is likely to be more successful in allowing them to retrieve a floating food reward. However, as with the developmental data, debate remains as to *what* is learned by non-human animals performing these experiments.

Previous research suggests that birds’ learning in the Aesop’s fable task is at an equivalent level to children of around 5-7-years [3]. However, direct comparison of performance is limited by differences in methodology and analysis between inter-species studies. As Miller and colleagues [23] observe, children tend to be given 3–5 trials in which to learn, and are assessed at the level of the age group, while birds tend to be given 15–20 trials, and are assessed at the level of the individual. When corvid and child data are analysed in the same way, birds appear to learn at a speed most redolent of 5-year-olds [8]. However, as reviewed above, it is not clear what type of information is required for learning over several trials. Furthermore, two individuals learning at the same rate does not imply they necessarily acquire the same information. While it may be the case that children do not solve the task using associative reward-based learning, it remains true that the task could *in principle* be solved using such an approach. Children and birds may well solve the same task, at the same speed, but in completely different ways.

In order to relate the current findings to corvids, it would be necessary to carry out an equivalent study within that population. Miller and colleagues [23] conducted a similar experiment, using a cut-down design that included only one of the F+R- (leaky) and F-R+ (syringe) conditions. In the exploration phase, birds were given 30 training trials with these object-context combinations before being presented with the same objects and a “standard” (unmodified) water tube in the choice phase. Over 5 choice trials, the birds picked significantly more non-functional, previously rewarded tools than functional, previously unrewarded tools, suggesting that they were responding more to reward history than functionality. However, the F+R-/F-R+ choice is the most challenging distinction, with a forced choice between function and reinforcement. As such, during training, the choice of a functional object would never have led to a reward. This would make it highly unlikely for the birds to adopt a function-based strategy even were they capable of doing so. Furthermore, this experiment took place immediately after another study in which birds received extensive training linking similar non-functional tools with reward. The birds were also given considerably more training than the children during the exploration phase. As discussed above, it is not known how this element may influence the development of different learning different types of information. Nonetheless, these findings may suggest that corvids may be more likely to solve the Aesop’s fable task using reward history.

### Caveats and limitations

As with any empirical study, interpretation of the current results rests upon several assumptions. First of all, how much the current findings can be generalised will depend on the degree to which the children assessed can be considered representative of a larger sample, and are comparable to those previously tested on other causal reasoning tasks. Another particular source of concern when dealing with any population, but particularly with school children, is the manner and extent to which previous learning plays a role in task performance. In particular, there is a concern that the performance of children of a certain age stems from having been actively taught water displacement during science lessons. This potential confound makes it particularly difficult to provide direct comparisons between children and non-human species. While it is possible, and in fact highly likely, that children are taught about water displacement at some stage of the school career, it is improbable they have directly associated this phenomenon with the experience of being rewarded. Rather, the choices that children would be likely to make by relying only on the knowledge that they may have previously learned about sinking and floating objects are predicted by the causal link model. This model however was never the best predictor of children’s choices, no matter the age. Were they to rely on academic knowledge to pass these tasks, one would also expect children at a particular point of progression in the school curriculum (which was shared by all participants in this study) to suddenly display a marked difference in the way they perform. However, subjects’ reliance on functionality shows a relatively steady progression with increasing age.

As with any cognitive test, performance on the Aesop’s fable task is likely to differ according not only to individual variation in the cognitive ability of interest, but also in overarching skills. ‘Executive function’ is an umbrella term encompassing a number of related abilities including inhibitory control, working memory and the “organisational strategies necessary to prepare a response” ([35], p.283). Executive functions exhibit a protracted development throughout childhood and adolescence (e.g. [36]), with preschool-age children struggling in particular with inhibitory control and response inflexibility. Difficulties with inhibition have been previously discussed in the context of the Aesop’s fable task as a possible limiting factor in performance in corvids relative to children [23]. An individual with poor or immature inhibitory control may have sufficient cognitive understanding to choose the correct action or tool, but fail to behave appropriately due to difficulty refraining from a prepotent response. Such an account may be relevant not only to differences in performance between children and non-human animals, but between children of different ages. In the current study, it is possible that the 4-year-olds did not differ from random or fit with a particular model not due to a lack of understanding but because of their well-documented difficulty in inhibiting impulsive responses [37, 38].

Another challenge to executive function in the present study was the need to bring together information from multiple sources to guide decision-making. Perner and colleagues [39] argue that 3-4-year-old children’s tendency towards inflexible behaviour in cognitive tasks may stem from a failure to understand that a stimulus or situation can be considered from different perspectives or according to different dimensions. As such they may find it difficult to integrate or separate information about functionality and reward in order to guide decision making, despite potentially understanding each element individually. Finally, the paradigm used in this experiment may have put particular demands on working and long-term memory. Children were required to remember a number of experiences with different objects and containers during the exploration phase in order to make the appropriate decision during the choice phase. While every effort was made to reduce this demand, such as representing the objects, containers and reward with picture cards throughout the study, it is still possible that this memory demand may have disproportionately affected younger children. Memory difficulties may therefore have limited the degree to which 4-5-year olds were able to use the available information in their choices.

While limitations in executive functions may explain why the youngest children in the sample did not demonstrate behaviour consistent with any model, it cannot account for the types of information used by the older children. It is very unlikely that failures of inhibition, information organisation or working memory would bias children’s responding in the direction of functionality-based decision making. More likely, issues with executive function, and inhibition in particular, would be likely to bias an individual towards reward-based associative learning, which makes fewer demands on executive functions. This account may explain the apparently reward-based responding seen in corvids on a comparable experiment [23].

Finally, the utility of these findings is dependent on the degree to which Aesop’s fable tasks are representative measures of physical cognition, and to what extent such tasks–or indeed tool-use tasks in general—are representative tests of causal learning as a broader concept. As such, a triangulation approach using a range of tasks and paradigms would be useful to understand how causal learning progresses in different tasks, and from a domain-general perspective. The area of social cognition may provide many relevant insights. Here too, the mechanistic association between cause and effect can be manipulated independently from reward by the use of scenarios in which a reward *would have* been achieved had the context been different. Call and colleagues [40] investigated whether apes were able to distinguish between the intentions of different human keepers even if the result of their behaviours were matched. The authors manipulated the behaviour of human keepers such that both denied the chimpanzees food, but one did so through “clumsiness” while the other intentionally withheld their gifts. They found that while neither keeper had actually given the animal food, chimpanzees showed preference for the individual who seemed like they *would have* given food had circumstances played out differently, i.e. if the context had been different. It would be theoretically possible to adapt such a design to explore comprehensively the relative contribution of functionality and reward-based learning to social causality.

In summary, our findings suggest that children’s choices on the Aesop’s fable task can be modelled using reward and function information from around the age of 5, years of age and that from the age of around 8 functionality-based models provide the best fit for children’s behaviour. By the time children reach the age of 10 models exclusively emphasising functionality information outstrip all other models. Models emphasising reward-history did not explain children’s choice behaviour at any age. These findings are in line with previous research suggesting that children begin to consistently pass complex tool-use tasks at around the age of 8, but require several trials in order to learn before this time. While some children, particularly in older age groups, relied exclusively on functional information, many children did not, suggesting that the influence of functional information may be more in the form of a decision-bias, rather than an exclusive strategy. Given that previously literature suggests that children within the age range tested *can* use associative learning of reward associations, the fact that they emphasise functionality information may suggest that children develop a tendency to seek and emphasise functionality information when learning in new situations, biasing their choices. Whether this tendency is the result of a natural bias or education is not clear, however it is plausible that such a bias would be vital in facilitating the development of adult-level causal reasoning.

## Supporting information

S1 File Raw data supporting analysis. (XLSX)
